# Striking forest revival at the end of the Roman Period in north-western Europe

**DOI:** 10.1038/s41598-020-77253-1

**Published:** 2020-12-15

**Authors:** C. Lambert, A. Penaud, M. Vidal, C. Gandini, L. Labeyrie, L. Chauvaud, A. Ehrhold

**Affiliations:** 1 CNRS, UMR 6538 Laboratoire Géosciences Océan (LGO), Univ. Brest (UBO), 29280 Plouzané, France; 2 UMR 6538 Laboratoire Géosciences Océan (LGO), Univ. Vannes (UBS), 56000 Vannes, France; 3 Centre de Recherche Bretonne et Celtique (CRBC), Univ. Brest (UBO), Brest, France; 4 AOROC-UMR 8546, CNRS, École Normale Supérieure, Paris, France; 5 CNRS, IRD, UMR 6539 Laboratoire des Sciences de l’Environnement Marin (LEMAR), Univ. Brest (UBO), 29280 Plouzané, France; 6 Géosciences Marines, Centre de Brest, IFREMER, Plouzané, France

**Keywords:** Climate sciences, Ocean sciences, Planetary science

## Abstract

The Holocene period (last 11,700 years BP) has been marked by significant climate variability over decadal to millennial timescales. The underlying mechanisms are still being debated, despite ocean–atmosphere–land connections put forward in many paleo-studies. Among the main drivers, involving a cluster of spectral signatures and shaping the climate of north-western Europe, are solar activity, the North Atlantic Oscillation (NAO) varying atmospheric regimes and North Atlantic oceanic gyre dynamics. Over the last 2500 years BP, paleo-environmental signals have been strongly affected by anthropogenic activities through deforestation and land use for crops, grazing, habitations, or access to resources. Palynological proxies (especially pollen grains and marine or freshwater microalgae) help to highlight such anthropogenic imprints over natural variability. Palynological analyses conducted in a macro-estuarine sedimentary environment of north-western France over the last 2500 years BP reveal a huge and atypical 300 year-long arboreal increase between 1700 and 1400 years BP (around 250 and 550 years AD) that we refer to as the ‘1.7–1.4 ka Arboreal Pollen rise event’ or ‘1.7–1.4 ka AP event’. Interestingly, the climatic 1700–1200 years BP interval coincides with evidence for the withdrawal of coastal societies in Brittany (NW France), in an unfavourable socio-economic context. We suggest that subpolar North Atlantic gyre strengthening and related increasing recurrence of storminess extremes may have affected long-term coastal anthropogenic trajectories resulting in a local collapse of coastal agrarian societies, partly forced by climatic degradation at the end of the Roman Period.

## Introduction

**Figure 1:**
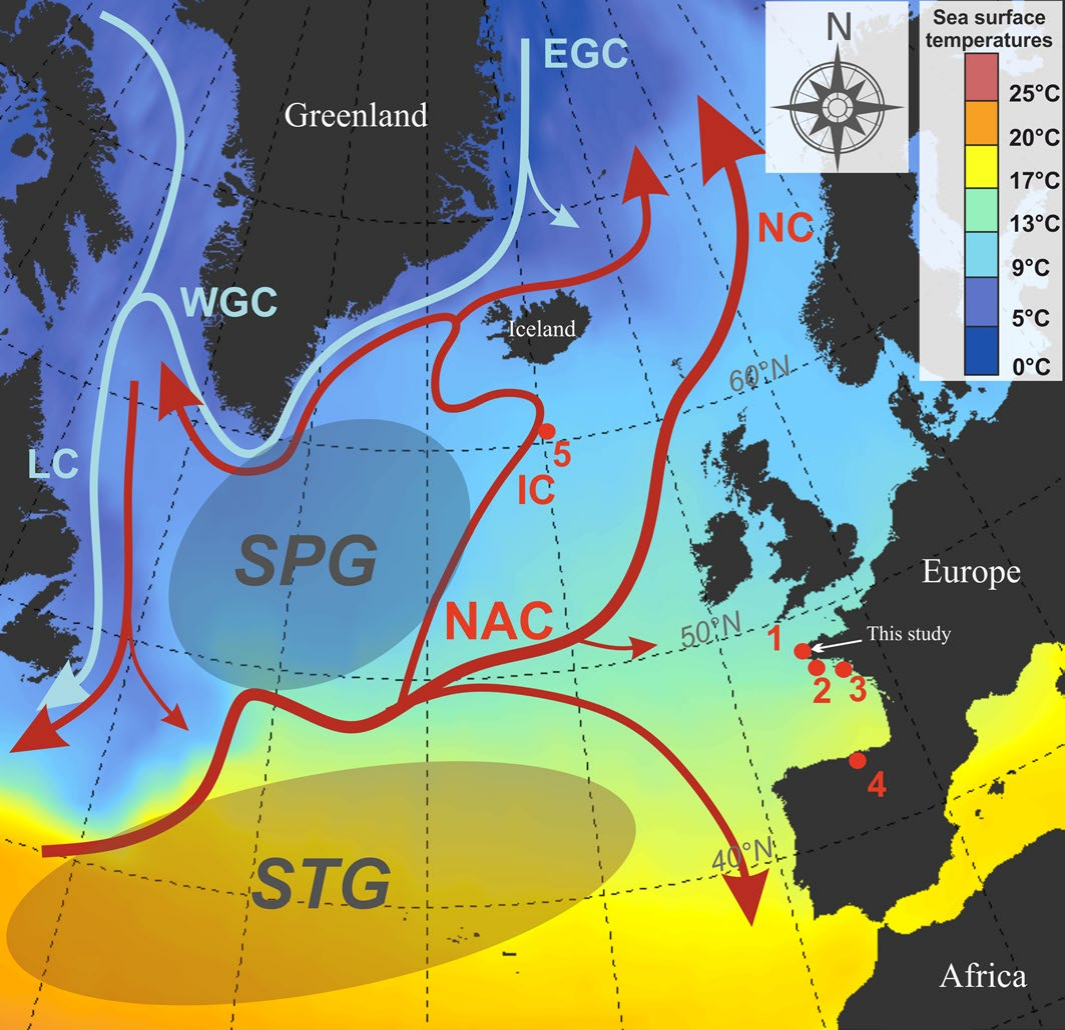
Map of Sea Surface Temperatures showing the schematic surface circulation of the North Atlantic Ocean. Red arrows characterize the main branches of the North Atlantic Current (NAC), namely the Irminger Current (IC) and the Norwegian Current (NC), bringing Subpolar Gyre (SPG) and Subtropical Gyre (STG) waters into the northern latitudes of the North Atlantic Basin. Blue arrows characterize colder surface waters, the East Greenland Current (EGC), the West Greenland Current (WGC) and the Labrador Current (LC). Sediment cores discussed in this study are represented by red dots: (1) study cores “G” and “KS-02” in the Bay of Brest, (2) core CBT-CS1 in the northern Bay of Biscay^21^, (3) core KV14bis at the Loire estuary mouth^33^, (4) Cueva de Asiul speleothem isotopic record in Northern Spain^34^, (5) core RAPID-12-1K south of Iceland^9^. The map was created using QGIS software version 3.4.5 (https://www.qgis.org).

**Figure 2:**
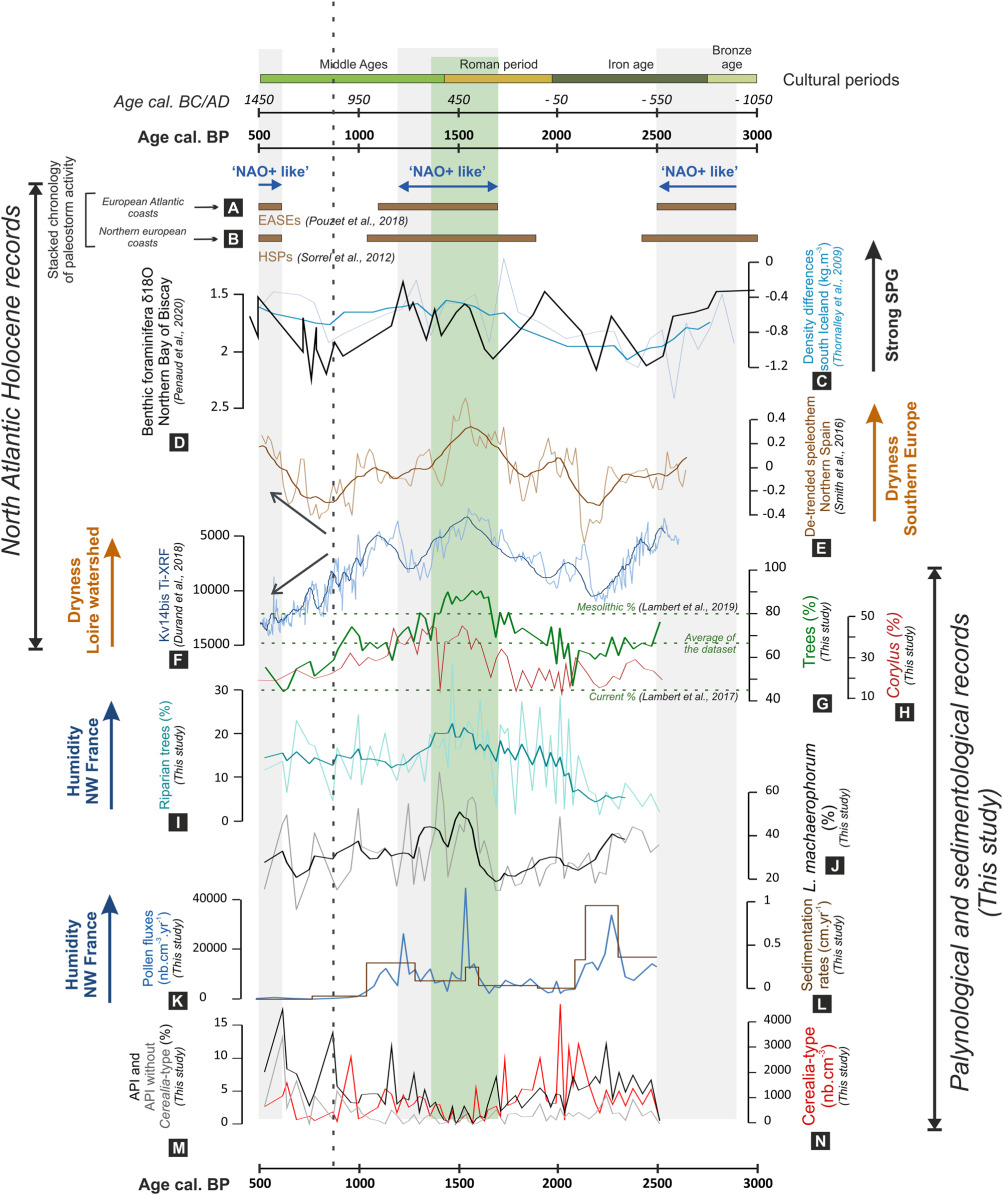
Comparison of palynological and sedimentological signals from the composite “G-KS02” Bay of Brest sequence (G–N) with paleoclimatic records from the North Atlantic: (A) European Atlantic storm events (EASEs) over European Atlantic coasts^23^, (B) Holocene storm periods (HSPs) over northern Europe^19^, (C) water density differences for SPG strength^9^, (D) δ18O on benthic foraminifera shells (*A. falsobeccarii*) from the core CBT-CS11 (Northern Bay of Biscay) for SPG strength^21^, (E) detrended and combined isotopic record from two Cueva de Asiul speleothems, Northern Spain^34^, (F) Ti-XRF record from core KV14bis at the Loire estuary mouth^33^, and main results from the composite “G-KS02” Bay of Brest sequence (this study): (G) sum of tree pollen percentages, (H) *Corylus* percentages, (I) sum of riparian tree (*Alnus, Salix* and *Betula*) percentages, (J) percentages of the dinocyst *Lingulodinium machaerophorum*, (K) total pollen grain fluxes, (L) sedimentation rates, (M) percentages of Anthropogenic Pollen Indicators (API: Asteraceae, Brassicaceae, *Fagopyrum*, *Plantago lanceolata*, Poaceae, *Polygonum aviculare*, *Rumex*) with (black) and without (grey) *Cerealia*-type, (N) *Cerealia*-type pollen concentrations. Light grey bands highlight intervals likely characterized by “NAO + like” atmospheric configuration (i.e., enhanced storminess and northward positioning of the westerly belt). The green band highlights the atypical forest cover increase in the Bay of Brest watersheds between 1700 and 1400 years BP. Dotted black line and grey arrows underline the divergence observed since 800 years BP between higher Loire runoff and lower precipitations reconstructed from northern Spain, likely pointing to an anthropogenic-related runoff forcing at that time.

The Holocene is a complex puzzle of climate variability^1–11^, characterized at a millennial-scale by a 2500-year solar-related cyclicity over the whole period, and the appearance of a 1500-year ocean-related cyclicity over the last 5000 years, i.e. during the Mid- to Late-Holocene^12^. Gyre dynamics in the Atlantic Ocean are considered as a primary mechanism controlling rapid climate changes, especially subpolar gyre (SPG) dynamics which modulate northward heat transport through the North Atlantic Current (NAC) as well as deep-water formation and sea-surface temperatures (SSTs) in the North Atlantic Basin^8–10^ (Fig. 1). Ocean surface density differences reconstructed south of Iceland and foraminifera shell isotopic analyses conducted in a core from the northern Bay of Biscay (Sites 2 and 5, Fig. 1) provide evidence of SPG strengthening intervals^9,11^ (Fig. 2C,D*)*, which led to a greater northward penetration of the warmer NAC in the northern Atlantic Ocean to the Nordic Seas^13–17^. Moreover, the North Atlantic Oscillation (NAO) pattern, defined as the difference between sea-level pressure over the Azores and Iceland, exerts strong control on the latitudinal position and vigour of the westerly wind belt^18^. An 'NAO-like' mechanism has been commonly evoked to explain paleoenvironmental evidence of climate variability over the Holocene^11,16,19,20^*.* Drift-ice discharge events^1^ corresponding to SPG strengthening phases^9,21^ would then correspond to recurrent positive modes of the NAO, as deduced from increasing storminess and moisture over northern Europe^16,19,22,23^.

North-western European coastal environments are interesting case studies because of their connection to the main Atlantic atmospheric and oceanic patterns that govern regional and global climates over different timescales. In this context, palynological studies can provide essential information about past environmental conditions through combined analyses of phytoplanktonic microalgae and pollen grains. High-resolution coastal records available close to continental sources such as watershed outlets are suitable archives for exploring climate-society relationships through time. Indeed, human–environment interactions remain poorly understood for pre-industrial periods, although the study of these times would allow us to put contemporary changes into perspective and thus improve understanding of underlying natural and/or anthropogenic forcings. The purpose of the present study is thus to identify past environmental and climate changes in a macro-estuarine sedimentary environment of north-western France (north-eastern Atlantic Ocean) over the last 2500 years BP and to discuss their potential implications for the dynamics of coastal societies. This study is relevant for north-western Europe but also for the wider understanding of long-term socio-ecosystemic trajectories.

## Results and discussion

Two highly temporally resolved sediment cores retrieved from the Bay of Brest (BB; Site 1, Fig. 1; Supplementary information Fig. 1) are used to infer crossed land-sea changes regarding vegetation dynamics on watersheds and sea-surface changes over the last 2500 years BP. We analysed pollen and dinoflagellate cyst (dinocyst) assemblages on a composite recording, including the 'G' core (from 2530 to 1660 years BP; 48°19′14″ N; 4°23′5″ W; 7.4 m depth; '*Défis Golfe de Gascogne*' cruise) and 'KS-02’ core (from 1660 to 500 years BP; 48°18′46″ N; 4°24′27″ W; 8 m depth; '*EssCalico*' cruise), with a temporal sampling resolution of about 35 years (Supplementary information Fig. 2). Over the last 2500 years BP, pollen results from the BB record show a great variability, with tree pollen percentages ranging between 45 and 90% (Fig. 2G). While tree percentages of 45% characterize the average values recorded in modern BB sediments^24^ (dotted line on Fig. 2G), the maximal values reached between 1700 and 1400 years BP led us to refer to this interval as the '1.7–1.4 Arboreal Pollen rise Event' or '1.7–1.4 ka AP event' (green band on Fig. 2G). Tree percentages exceed even those values recorded close to the studied area during the Mesolithic at around 9000 years BP^25^ (~ 80%; dotted line on Fig. 2G), which is a period characterized by few or no major human-forced environmental changes. The ‘1.7–1.4 ka AP event’ is all the more striking as it takes place in a major increasing phase of landscape opening (detectable in pollen sequences since around 4000 years BP^11,26^). This atypical arboreal pollen increase, only noticed in a few records^11,26,27^, thus raises the question of its climate and/or anthropogenic origin (i.e. agricultural abandonment, probably leading to a forest revival).

Between 1700 and 1400 years BP, the mixed oak forest peak, mainly supported by *Corylus* in the BB (Fig. 2H), could be partly attributed to moister and milder conditions in north-western Europe. During the 1.7–1.4 ka AP event, humid conditions are supported by increases in riparian pollen percentages as *Alnus* and *Salix* (Fig. 2I), the latter being recently discussed as powerful fluvial discharge tracers in the studied area^11,21,24,28^. Temporary increases of total pollen fluxes during the climatic interval from 1700- to 1200 years BP also testify to the increase in fluvial terrigenous inputs, which further enhanced the transport of pollen grains (Fig. 2K). Continental evidence of moisture increase is also corroborated by dinocyst observations (Fig. 2J). *Lingulodinium machaerophorum*, an estuarine-proliferating species^29,30^ that tolerates strong drops in salinity^29,31^, testifies to coastal stratified waters subject to strong continental influence^11,21^ and, thus, to stronger fluvial discharges at the studied site. Humid conditions together with an increase of fluvial inputs may explain the *Corylus* pollen rise during the 1.7–1.4 ka AP event in the BB. Moreover, this interval is also characterized by wetter conditions over northern central Europe then reflecting an eastward extension of the climatic conditions reconstructed in the BB^32^.

Recent studies conducted over south-western Europe allow us to enlarge our observations. In the Bay of Biscay (Site 3, Fig. 1), a large fall in Titanium-XRF counts (Ti-XRF; Fig. 2F) was observed between 1700 and 1400 years BP in a sediment core retrieved at the outlet of the Loire River^33^. This signal was interpreted as a result of a smaller contribution of Loire River discharges and thus of lower runoff over Loire watersheds^11,33^. The decrease in markers of fluvial influence recorded in a marine core from the northern Bay of Biscay (Site 2, Fig. 1) also testify to the decrease of the Loire contribution^11,21^ during the 1.7–1.4 ka AP event in the BB. Furthermore, between 2700 and 800 years BP, precipitation quantifications carried out in speleothems of northern Spain^34^ (Site 4, Figs. 1; 2E; Cueva de Asiul) show comparable variations to those of Loire paleo-fluvial discharges with a decrease of moisture between 1700 and 1300 years BP^33^. An ‘aridification event’ was also detected in the southwestern Mediterranean area, characterized by forest retraction and a decrease in fluvial inputs^35–37^, suggesting similar hydro-climatic influences on southern European sites. We suggest that the obvious correlation between higher humidity indexes in the BB (tree pollen percentages, especially *Corylus* and *Alnus*, as well as *L. machaerophorum* dinocysts; Fig. 2G,I,J) and higher aridity indexes to the south (lower Loire terrigenous inputs to the sea, Fig. 2F; Mediterranean aridity markers^35–37^), could attest to the presence of a differential north–south behaviour similar to the one described by the NAO pattern today, responsible for contrasting weather and precipitation regimes over Europe^18,38^. After 800 years BP (black dotted line on Fig. 2), the Ti-XRF signature from the Loire marine core (Fig. 2F) and precipitations reconstructed in northern Spain (Fig. 2E) show the reverse pattern compared with that described above. From this threshold, the Ti-XRF terrigenous detrital proxy for Loire watersheds seems to become a marker of soil erosion caused by massive human deforestation following the Medieval Climatic Optimum^11^, rather than a paleo-fluvial proxy in a context of enhanced precipitations. Tree percentages in BB watersheds also sharply decline at that time and reach values below the average of the 2500 year-long dataset, pointing to a pronounced anthropogenic limit for the Late Holocene in the studied area (green dotted line on Fig. 2G).

**Figure 3:**
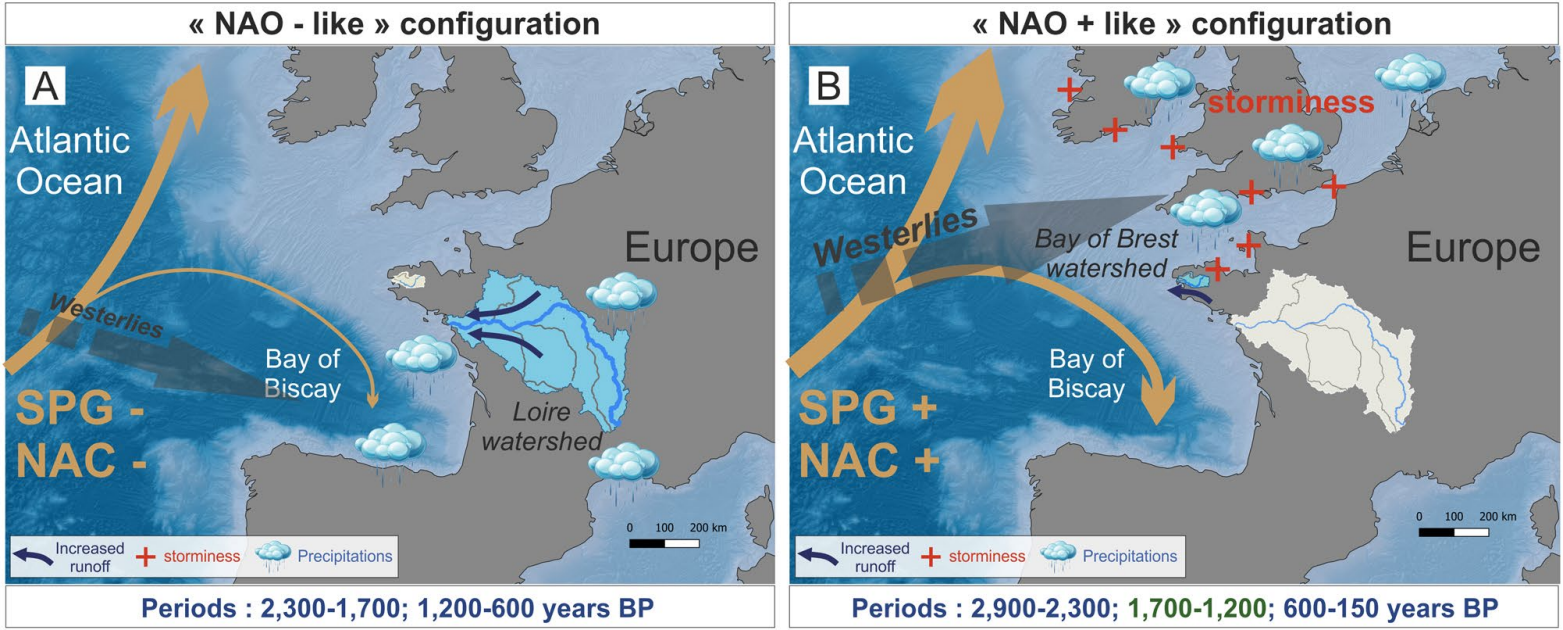
Schematic conceptual models showing storm tracks positioning, North Atlantic Current (NAC) and Subpolar Gyre (SPG) vigor during (**A**) ‘NAO + like’ and (**B**) ‘NAO− like’ pluri-secular intervals and the associated influence on storms and precipitation patterns over North West Europe. Watersheds in blue represent increased precipitation and runoff. The map was created using QGIS software version 3.4.5 (https://www.qgis.org).

These data therefore allow us to consider a climatic hypothesis for the 1.7–1.4 ka AP event. We assume that a reinforced SPG and the subsequent intensification of the NAC, promoting northward oceanic heat transport and SPG-related internal climate feedback (e.g., reduced winter sea-ice coverage, near-surface atmosphere warming, negative sea-level pressure leading to a northward positioning of the westerly belt; Fig. 3), are the main drivers for the warm, moist winters recorded at the end of the western European Roman Period, thus explaining the temperate forest growth.

In the climatic context of increasing fluvial discharges and pollen fluxes (Fig. 2K,L), the major peak of trees (exceeding the Mesolithic rate) is, moreover, observed synchronously with a sharp decrease in cultivated plants (*Cerealia*-type pollen grain concentrations, Fig. 2N, as well as anthropogenic pollen indicators, API, Fig. 2M; Supplementary Fig. S3). We thus hypothesize that the 1.7–1.4 ka AP event (Fig. 2, green band) may also reflect a local collapse of the agrarian system allowing reforestation of wetlands and abandoned agricultural plots especially around 1500 years BP. The end of the Roman Period was also perturbed by political, economic, social and military unrests^39,40^, which could have been locally exacerbated by the increased storminess known on a broader coastal European-scale, during the 1700–1200 years BP climatic interval^23^ (Fig. 2A) or the larger 1900–1050 years BP interval^19^ (Fig. 2B). From an archaeological point of view, fine ceramics from this period are rare and little known coupled with poor conservation of late levels. This bias can indeed hamper identification of the occupation phases of the late Roman Period^41^. For the best documented regions in north-western Europe (e.g., the centre of France^42,43^), the fifth century (i.e., after 1600 years BP) shows a sharp reduction in the number of occupied sites, while the creation of settlements seems to represent a marginal phenomenon. Nevertheless, the interpretation of this trend is still widely discussed as the rate and extent of withdrawals can vary considerably by region. Moreover, studies have shown changes since the beginning of the second century (i.e. around 1850 years BP), well before the main upheavals of the 3rd–4th (i.e. between 1750 and 1500 years BP) centuries, urging caution regarding the explanation of military disturbances and suggesting rather a gradual reorganisation of land use and the production system^42,44^. Thus, we suggest that the climatic degradation, which could for example have caused significant floods, could have been an amplifying factor in the collapse of the otherwise struggling agrarian society in the region.

A combination of oceanic (i.e., SPG and NAC strengthening) and atmospheric configurations (i.e., northward position of the westerly belt), strongly influence European climate over the Late Holocene (Fig. 3). The 1.7–1.4 ka AP event may result from both greater humidity and major phases of human withdrawals in a general context of regional climatic deterioration. Our pollen records thus agree with a 300-year-long collapse of coastal agrarian societies recorded in NW France and probably partly forced by climatic variability. At 800 years BP, a resumption of deforestation activities contributed to a landscape opening rate similar to that reconstructed before the 1.7–1.4 ka AP event.

## Methods

### Stratigraphy of the composite G-KS-02 study core

Both studied cores (Supplementary Table S1) were taken in the Bay of Brest macro-estuary (NW France; Supplementary Fig. S1) onboard the RV ‘Côtes de la Manche’ in the framework of (i) the Ifremer program 'Défis Golfe de Gascogne' (2003, vibrocorer) for G core and (ii) the 'EssCALICO' cruise (2010, gravity corer) for the Ks-02 core. Descriptions, photographs and X-ray radiographies are available (Supplementary Fig. S1). A total of 14 radiocarbon dates were acquired on gastropods (*Turritella* spp., *Caliostoma zizyphinum*, *Bittium reticulatum*) for the G core (six ^14^C-AMS dates) and the KS-02 core (eight 14C-AMS dates). All 14C-AMS dates (Supplementary Table S2) were calibrated to calendar years with the CALIB 7.1 program using the Marine13 calibration curve^45^ which considers a 400-year correction for the mean ocean surface reservoir age. Reservoir age can, however, vary spatially and temporally. The local deviation from the oceanic mean (ΔR) is estimated at − 40 ± 23 years off the Brittany peninsula^46^, as confirmed by a reservoir age test in the BB^25^. We applied this additional correction to all dating obtained on marine carbonate material. Thus, all the dates were calibrated and are expressed hereafter in calendar age (Cal.) BP (‘before present’, before 1950).

The age model produced on the KS-02 core confirms that this sedimentary sequence provides information on the continuity of the very closed G core (whose upper part was missing), thus making it possible to constitute a long composite sequence, as illustrated by the continuity in the pollen data (Supplementary Fig. S3). The composite age model was established with the 'Bacon' age-depth modelling package^47^ in R software (Supplementary Fig. S2) and makes it possible to document a period of around 2000 years (from 2528 to 488 years Cal. BP). In the manuscript, when referring to a precise age, ‘Cal.’ will not be systematically written, and ages will often be specified as ‘year BP’ or ‘year BC’ to facilitate the reading of results by paleoenvironmental or archaeological communities, respectively.

### Palynological analyses

In this study, we analysed 58 samples (27 for core G, 31 for core KS-02). Palynological preparations on the < 150 µm sediment fraction were carried out at the EPOC laboratory (University of Bordeaux, France). The procedure is based on chemical (cold 10–25–50% HCl and cold 40–70% HF) treatments to remove siliceous and carbonate fractions, as well as physical sieving (10-µm nylon mesh screen) to remove the mineral clayey fractions and concentrate organic micro-fossils (i.e., palynomorphs)^48^. Final residues were mounted with glycerine between a slide and a coverslip. Pollen and dinocysts were identified using an optical microscope Leica DM2500 at X630 magnification^49–51^.

For each sample, a minimum of 300 pollen grains and 150 dinocysts were systematically counted (Supplementary Table S3) in order to provide robust assemblages from a statistical point of view^52^. Percentages were calculated on a total pollen or total dinocyst sum with no taxon exclusion, and concentrations were expressed in number of palynomorphs/cm^3^ using the ‘marker grain method’, which consists in adding a known number of exotic spores (*Lycopodium clavatum*) to dried sediments before any palynological treatments^53^.

## Supplementary information

Supplementary Information.

## Data Availability

The datasets generated during this study are partly included in this published article and its supplementary information and are available from the corresponding author upon reasonable request.
