# Supplementary material for: Striking forest revival at the end of the Roman Period in north-western Europe
Source: Sci Rep. 2020 Dec 15;10:21984. doi: 10.1038/s41598-020-77253-1 (PMC7738505; doi:10.1038/s41598-020-77253-1)
Supplement: Supplementary file 1 — Supplementary Information. [file 41598_2020_77253_MOESM1_ESM.docx]

Striking forest revival at the end of the Roman Period in north-western Europe

Supplementary material

*Lambert C.^1,2*^, Penaud A.^1^, Vidal M.^1^, Gandini C.^3,4^, Labeyrie L.^2^, Chauvaud L.^5^, Ehrhold A.^6^*

^1^ Univ. Brest (UBO), CNRS, UMR 6538 Laboratoire Géosciences Océan (LGO), F-29280 Plouzané, France

^2^ Univ. Vannes (UBS), UMR 6538 Laboratoire Géosciences Océan (LGO), F-56000 Vannes, France

^3^ Univ. Brest (UBO), Centre de Recherche Bretonne et Celtique (CRBC), Brest, France

^4^ AOROC - UMR 8546, CNRS, École Normale Supérieure, Paris

^5^ Univ. Brest (UBO), CNRS, IRD, UMR 6539 Laboratoire des Sciences de l’Environnement Marin (LEMAR), F-29280 Plouzané, France

^6^ Géosciences Marines, Centre de Brest, IFREMER, France

**Environmental setting**

The Bay of Brest (BB), located in north-western Brittany (NW France, NW Europe) is a shallow semi-enclosed basin of 180 km² surrounded by a 230 km long coastline. During the Quaternary, the BB was subjected to alternations of glacial lowstands and interglacial highstands; the last transgressive episode thus corresponded to a Holocene sedimentary infilling of the BB^1^.

Today, the BB is characterized by a macrotidal influence. Grain-size analyses performed on modern BB sediments reflect specific hydrodynamic conditions mainly related to tidal currents^2^. In the stream direction, sedimentation is mainly composed of sands and gravels^2^. On either side of this axis, the current speed is strongly reduced and grain-size is characterized by fine to very fine sediments (silts and clays)^2,3^. In its easternmost part, the BB receives its main freshwater supplies from the Aulne and the Elorn (Supplementary Fig. S1) whose outflows contribute to up to 85% of the total river discharges into the BB^4^.

**Modern climate**

The Brittany coasts, located in the north-eastern Atlantic, are subject to a temperate oceanic climate. The vicinity of the ocean carries humid air masses from westerly winds (westerlies) to them throughout the year and low thermal seasonal amplitude is recorded between winter and summer months. Despite moderate inter-annual variations in temperatures, mean annual values are about 10- 11°C^5^. Annual prevailing winds mainly have a southwestern origin. Since Brittany is subject to regular oceanic rainfall, annual cumulative precipitation ranges from 600 to more than 1,600 mm/year in the inner part^5^. Moreover, the climate of Brittany is created by the combined influences of fluctuating atmospheric (North Atlantic Oscillation, NAO) and oceanic (AMOC and gyre dynamics) patterns^6,7^.

The NAO is the major atmospheric circulation pattern controlling climatic variability in western Europe. It affects wind, precipitation and winter temperatures^8^. Positive (/negative) phases (NAO +; high gradient between sea-level pressure over Iceland and the Azores anticyclonic centre) are characterized by strengthened (/weakened) mid-latitude westerlies over northern Europe, giving rise to a storm corridor over Brittany and to mild (/cold) and moist (/dry) winters^8,9^. Positive phases of the NAO are conventionally associated with above-average continental precipitation in northwestern Europe (i.e. north of the Loire watershed) and with droughts further south^6,10^.

Moreover, on decadal to multi-decadal timescales, variations in North Atlantic SST appear to control much of the climate variability on the continent^7,11^. This oscillation is related to the energy (i.e. solar irradiance) received by intertropical water masses and its redistribution by major Atlantic Ocean currents^12^. A stronger (/weaker) NAC (North Atlantic Current) and SPG (subpolar gyre) lead to greater (/lesser) heat transport from the tropics to the north and higher (/lower) North Atlantic SST^7^. SST therefore impacts both rainfall and atmospheric temperatures^13,14^.

**Supplementary material references**

1. Gregoire, G., Le Roy, P., Ehrhold, A., Jouet, G., & Garlan, T. Control factors of Holocene sedimentary infilling in a semi-closed tidal estuarine-like system: the bay of Brest (France). *Mar. Geol.*, **385**, 84-100 (2017).
2. Gregoire, G., Ehrhold, A., Le Roy, P., Jouet, G., & Garlan, T. Modern morpho-sedimentological patterns in a tide-dominated estuary system: the Bay of Brest (west Britanny, France). *J. Maps*, **12**(5), 1152-1159 (2016).
3. Lambert, C. et al. Modern palynological record in the Bay of Brest (NW France): Signal calibration for palaeo-reconstructions. *Rev. Palaeobot. Palynol.*, **244**, 13-25 (2017).
4. Delmas, R., & Treguer, P. Evolution saisonnière des nutriments dans un écosystème eutrophe d'Europe occidentale (la rade de Brest). Interactions marines et terrestres. *Oceanol. acta*, **6**(4), 345-356 (1983).
5. Belleguic, K., Conseil, C., Eveno, T., Lorge, S. & Baraer, F. *Le Changement climatique en Bretagne* (ed. Météo France)*.* 85 pp (Paris, 2012).
6. Tréguer, P., et al. Large and local-scale influences on physical and chemical characteristics of coastal waters of Western Europe during winter. *J. Marine Syst.*, **139**, 79-90 (2014).
7. Ruprich-Robert, Y., & Cassou, C. Combined influences of seasonal East Atlantic Pattern and North Atlantic Oscillation to excite Atlantic multidecadal variability in a climate model. *Climate Dyn.*, **44**(1-2), 229-253 (2015).
8. Hurrell, J. W., Kushnir, Y., Ottersen, G. & Visbeck, M. in *North Atlantic Oscillation: Climate Significance and Environmental Impact* (eds Hurrell, J. W., Kushnir, Y., Ottersen, G. & Visbeck, M.) 1–35 (Geophys. Monogr. 134, American Geophysical Union, 2003).
9. Morley, A., Rosenthal, Y., & DeMenocal, P. Ocean-atmosphere climate shift during the mid-to-late Holocene transition. *Earth Planet. Sci. Lett.*, **388**, 18-26 (2014).
10. Rodriguez‐Puebla, C., Encinas, A. H., Nieto, S., & Garmendia, J. Spatial and temporal patterns of annual precipitation variability over the Iberian Peninsula. *Int. J. Climatol.*, **18**(3), 299-316 (1998).
11. Knight, J. R., Folland, C. K., & Scaife, A. A. Climate impacts of the Atlantic multidecadal oscillation. *Geophys. Res. Lett.*, **33**(17) (2006).
12. Knudsen, M. F., Jacobsen, B. H., Seidenkrantz, M. S., & Olsen, J. Evidence for external forcing of the Atlantic Multidecadal Oscillation since termination of the Little Ice Age. *Nature Comm.*, **5**(1), 1-8 (2014).
13. Kerr, R. A. A North Atlantic climate pacemaker for the centuries. *Science*, **288**(5473), 1984-1985 (2000).
14. Msadek, R., & Frankignoul, C. Atlantic multidecadal oceanic variability and its influence on the atmosphere in a climate model. *Climate Dyn.*, **33**(1), 45-62 (2009).

Supplementary Fig. S1 :


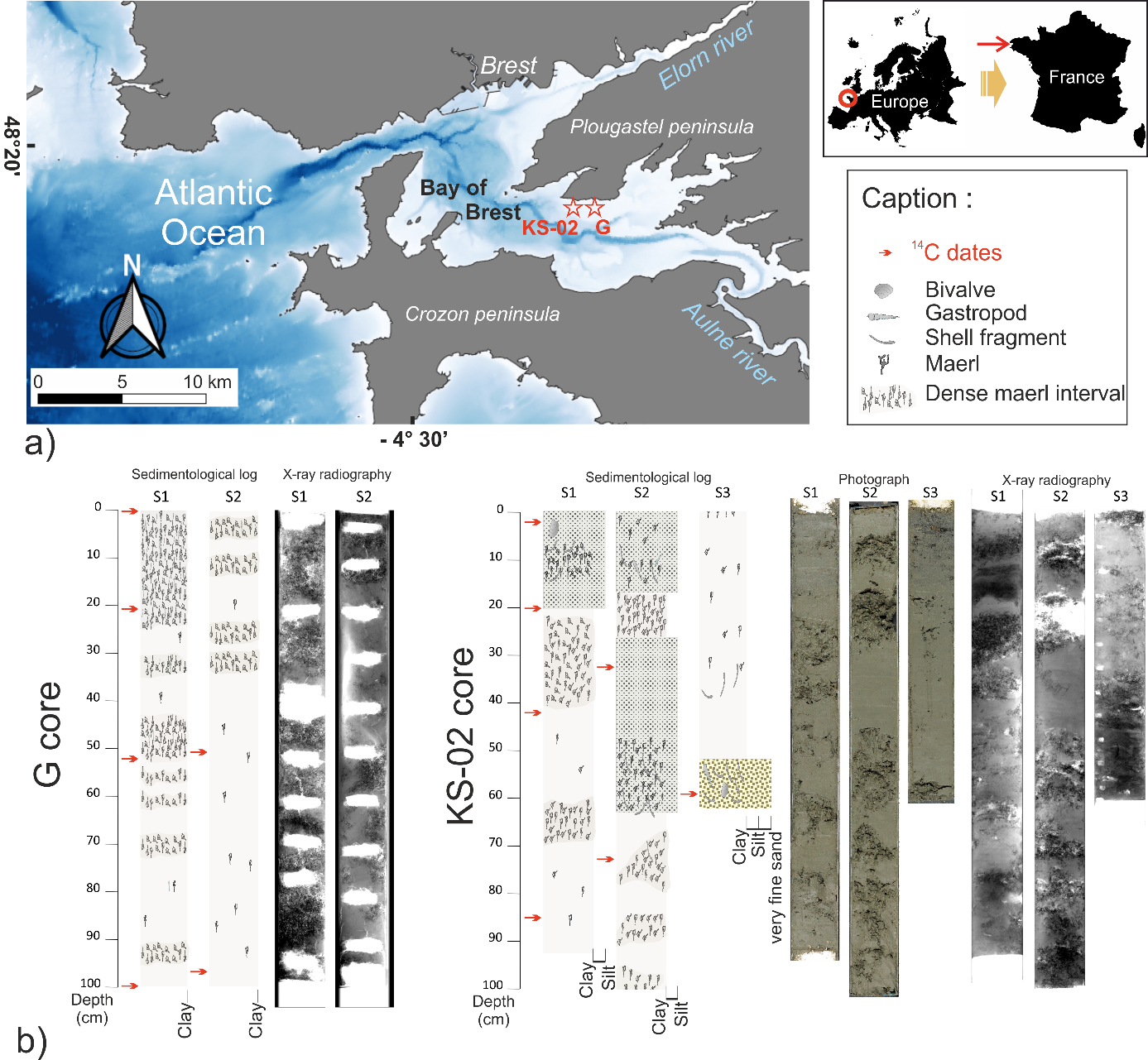


Supplementary Fig. S2 :


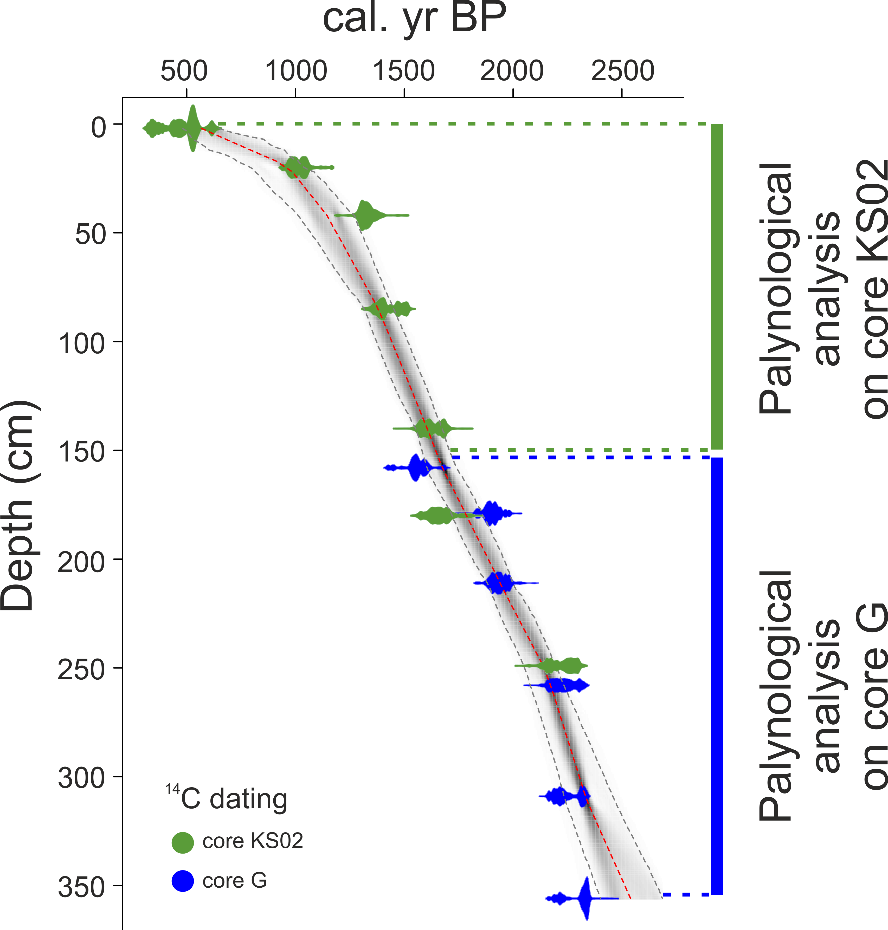


Supplementary Fig. S3 :


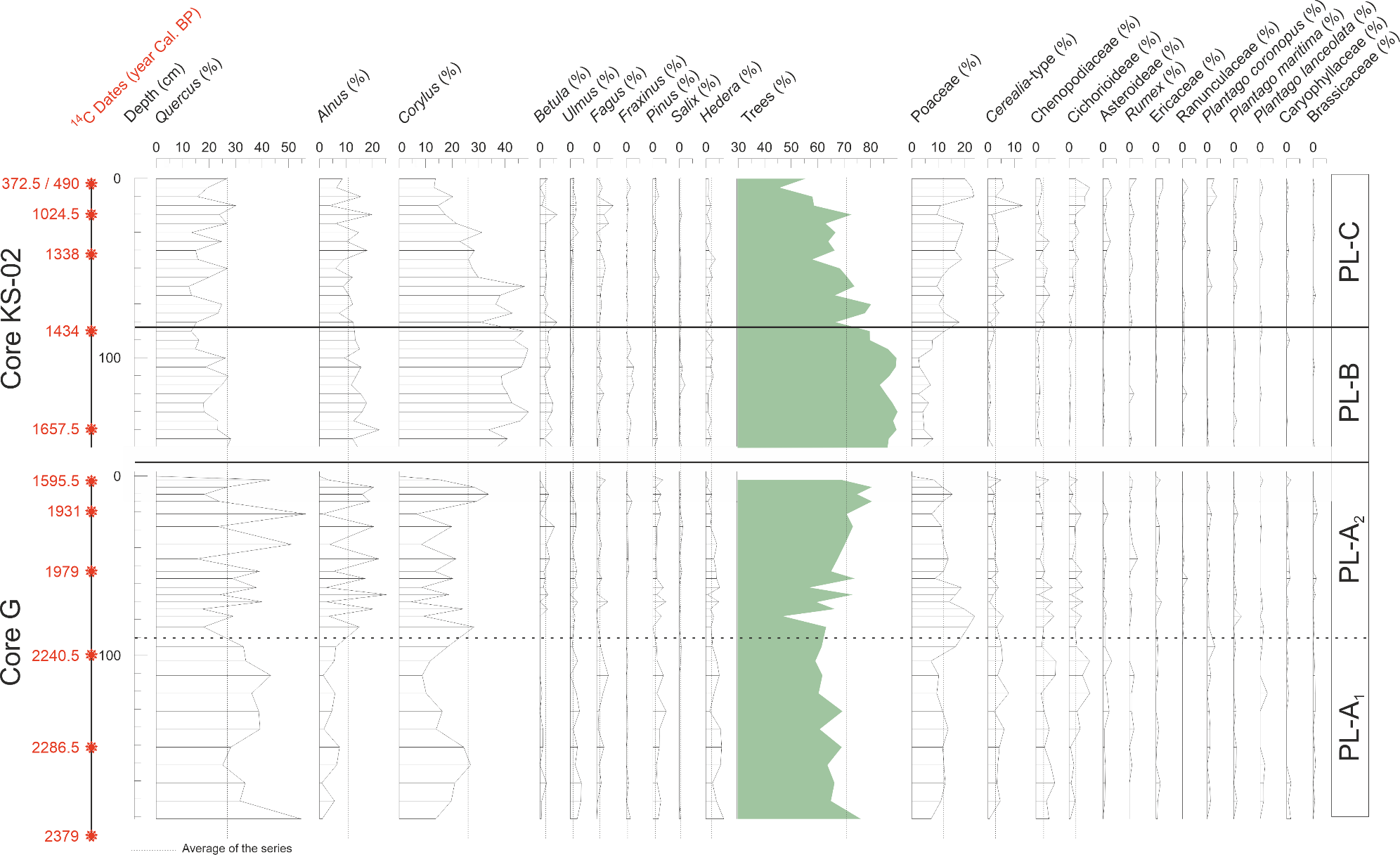


Supplementary Table S1:

| Name | Latitude | Longitude | Depth | Lenght | Coring date |
| --- | --- | --- | --- | --- | --- |
| G | 48°19’14’’ N | 4°23’5’’ W | 7.4 m | 358 cm | 2003 |
| EssCALICO-KS02 | 48°18’46’’ N | 4°24’27’’ W | 8 m | 253.5 cm | 2010 |

Supplementary Table S2:

| Lab code | Depth (cm) | material | Age ^14^C | Error | Age cal. BP | Age cal. AD/BC |
| --- | --- | --- | --- | --- | --- | --- |
| Poz-85159 | **2** | Gastropod | 690 | 30 | 289-456  **(372.5)** | **1577.5 AD** |
| SacA47759 | **2** | Gastropod | 835 | 35 | 420-560  **(490)** | **1460 AD** |
| SacA49423 | **20** | Gastropod | 1430 | 30 | 924-1125  **(1024.5)** | **925.5 AD** |
| SacA47761 | **42** | Gastropod | 1740 | 40 | 1238-1438  **(1338)** | **612 AD** |
| Poz-78153 | **85-86** | Gastropod | 1850 | 30 | 1338-1530  **(1434)** | **516 AD** |
| SacA45632 | **140-141** | Gastropod | 2040 | 30 | 1546-1769  **(1657.5)** | **292.5 AD** |
| Poz-78154 | **180-181** | Gastropod | 2075 | 35 | 1575-1814  **(1694.5)** | **255.5 AD** |
| SacA45633 | **249-250** | Gastropod | 2515 | 30 | 2124-2320  **(2222)** | **272 BC** |

**Core KS-02**

| **Lab code** | **Depth (cm)** | **Material** | **Age 14C** | **Error** | **Age cal. BP** | **Age cal. AD/BC** |
| --- | --- | --- | --- | --- | --- | --- |
| Poz-42846 | **0-1** | Gastropod | 1980 | 30 | 1494-1697  **(1595.5)** | **354.5 AD** |
| SacA45629 | **20-23** | Gastropod | 2275 | 30 | 1824-2038  **(1931)** | **19 AD** |
| Poz-73703 | **53** | Gastropod | 2310 | 30 | 1869-2089  **(1979)** | **29 BC** |
| Poz-42847 | **100** | Gastropod | 2545 | 35 | 2136-2345  **(2240.5)** | **290.5 BC** |
| Poz-73704 | **150-152** | Gastropod | 2580 | 30 | 2166-2407  **(2286.5)** | **336.5 BC** |
| Poz-42848 | **198.5** | Gastropod | 2625 | 30 | 2274-2484  **(2379)** | **429 BC** |

**Core G**

Supplementary Table S3:

| Age (Cal. BP) | Trees (%) | Corylus (%) | Riparian trees (%) | L. machaerophorum (%) | API (%) | API without Cerealia-type (%) | Cerealia-type (nb.cm^-3^) | |  |
| --- | --- | --- | --- | --- | --- | --- | --- | --- | --- |
| 521 | 55,409 | 13,720 | 11,609 | 16,062 | 7,916 | 2,639 | | 631,758 | |
| 612 | 44,292 | 14,612 | 13,699 | 46,154 | 17,352 | 13,242 | | 1061,704 | |
| 635 | 45,676 | 13,243 | 6,486 | 50,265 | 8,378 | 2,432 | | 1576,723 | |
| 680 | 54,645 | 13,661 | 22,951 | 6,897 | 7,650 | 6,557 | | 126,352 | |
| 748 | 58,065 | 20,276 | 17,512 | 22,535 | 2,765 | 0,000 | | 260,600 | |
| 771 | 51,337 | 13,904 | 17,112 | 41,935 | 4,813 | 4,278 | | 94,764 | |
| 864 | 58,687 | 14,672 | 4,633 | 20,833 | 13,900 | 0,772 | | 427,922 | |
| 887 | 63,571 | 25,714 | 16,786 | 15,789 | 5,714 | 5,000 | | 57,911 | |
| 956 | 73,881 | 17,413 | 9,453 | 24,706 | 3,234 | 1,741 | | 2606,000 | |
| 993 | 69,649 | 16,613 | 24,281 | 53,226 | 3,195 | 2,556 | | 163,514 | |
| 1009 | 63,056 | 21,667 | 8,333 | 35,833 | 4,444 | 1,667 | | 210,588 | |
| 1049 | 66,794 | 31,298 | 17,176 | 33,898 | 4,580 | 0,763 | | 434,333 | |
| 1089 | 64,080 | 22,989 | 12,644 | 32,824 | 4,598 | 0,575 | | 601,440 | |
| 1129 | 66,766 | 28,443 | 20,060 | 35,294 | 2,994 | 0,000 | | 1336,410 | |
| 1161 | 58,530 | 26,247 | 12,073 | 30,986 | 11,811 | 2,100 | | 1135,371 | |
| 1188 | 68,860 | 27,632 | 9,211 | 17,391 | 3,070 | 1,316 | | 397,105 | |
| 1217 | 71,820 | 29,925 | 15,461 | 34,320 | 3,990 | 0,000 | | 1096,851 | |
| 1244 | 73,947 | 47,368 | 11,053 | 55,385 | 3,684 | 0,526 | | 613,176 | |
| 1271 | 67,064 | 38,186 | 13,126 | 18,269 | 7,637 | 1,432 | | 747,964 | |
| 1299 | 80,240 | 36,527 | 15,569 | 35,417 | 1,796 | 0,000 | | 1051,160 | |
| 1326 | 77,861 | 42,786 | 8,955 | 19,892 | 5,224 | 0,995 | | 944,379 | |
| 1353 | 67,602 | 30,612 | 19,898 | 33,898 | 2,551 | 1,531 | | 272,523 | |
| 1379 | 80,124 | 46,998 | 16,977 | 43,210 | 3,313 | 0,828 | | 720,447 | |
| 1401 | 80,058 | 43,353 | 16,763 | 70,000 | 1,734 | 0,000 | | 714,789 | |
| 1422 | 86,910 | 48,706 | 19,178 | 55,046 | 0,609 | 0,152 | | 223,923 | |
| 1442 | 89,827 | 47,619 | 12,121 | 21,818 | 0,433 | 0,433 | | 0,000 | |
| 1463 | 84,169 | 7,916 | 35,884 | 30,645 | 2,639 | 1,055 | | 453,094 | |
| 1483 | 87,862 | 38,587 | 16,304 | 37,037 | 0,000 | 0,000 | | 0,000 | |
| 1504 | 85,638 | 39,007 | 16,667 | 45,714 | 1,064 | 0,355 | | 94,418 | |
| 1524 | 86,258 | 41,015 | 19,450 | 42,188 | 2,748 | 2,114 | | 279,214 | |
| 1544 | 88,298 | 42,553 | 22,766 | 56,881 | 1,489 | 0,426 | | 373,205 | |
| 1565 | 90,580 | 48,913 | 21,014 | 58,333 | 0,000 | 0,000 | | 0,000 | |
| 1585 | 88,550 | 45,802 | 15,725 | 55,422 | 0,763 | 0,000 | | 1380,857 | |
| 1605 | 90,275 | 33,827 | 27,696 | 32,530 | 1,268 | 0,423 | | 262,239 | |
| 1625 | 86,888 | 40,900 | 14,090 | 38,519 | 0,978 | 0,783 | | 69,917 | |
| 1644 | 87,546 | 36,630 | 20,879 | 33,333 | 2,930 | 0,733 | | 496,381 | |
| 1685 | 69,061 | 15,470 | 3,315 | 20,000 | 7,182 | 2,210 | | 657,841 | |
| 1705 | 80,488 | 28,293 | 21,951 | 15,556 | 1,951 | 0,976 | | 262,239 | |
| 1726 | 74,936 | 33,760 | 19,949 | 22,727 | 2,558 | 0,000 | | 2481,905 | |
| 1746 | 80,519 | 29,221 | 21,212 | 26,829 | 3,247 | 1,515 | | 694,933 | |
| 1781 | 70,968 | 6,452 | 1,613 | 19,355 | 4,301 | 1,613 | | 831,499 | |
| 1815 | 73,348 | 19,824 | 27,313 | 18,699 | 2,423 | 1,101 | | 1341,099 | |
| 1866 | 70,270 | 8,408 | 6,907 | 34,615 | 4,204 | 1,201 | | 1290,556 | |
| 1906 | 67,584 | 21,407 | 25,994 | 21,649 | 5,505 | 3,058 | | 2560,281 | |
| 1940 | 65,122 | 13,415 | 7,805 | 25,397 | 4,390 | 0,488 | | 887,594 | |
| 1961 | 73,987 | 20,256 | 19,616 | 27,612 | 2,559 | 1,279 | | 737,051 | |
| 1988 | 56,908 | 8,553 | 3,947 | 28,000 | 4,605 | 0,658 | | 768,993 | |
| 2007 | 73,311 | 18,750 | 28,378 | 49,275 | 4,392 | 2,365 | | 4710,104 | |
| 2028 | 59,364 | 4,594 | 2,827 | 21,905 | 1,767 | 1,060 | | 106,194 | |
| 2049 | 66,441 | 24,068 | 23,390 | 28,235 | 4,407 | 1,695 | | 2967,131 | |
| 2070 | 46,884 | 9,199 | 3,561 | 27,731 | 7,122 | 1,187 | | 1492,691 | |
| 2101 | 63,229 | 28,251 | 15,022 | 25,610 | 5,157 | 1,794 | | 3127,200 | |
| 2153 | 61,538 | 18,315 | 6,960 | 34,653 | 5,861 | 0,733 | | 1508,267 | |
| 2182 | 59,167 | 11,667 | 6,111 | 22,430 | 7,222 | 1,667 | | 758,531 | |
| 2207 | 61,818 | 8,727 | 1,455 | 26,000 | 6,182 | 2,545 | | 390,420 | |
| 2238 | 60,392 | 10,196 | 6,667 | 23,684 | 12,157 | 4,314 | | 1290,556 | |
| 2270 | 69,380 | 16,279 | 4,651 | 41,322 | 3,876 | 1,550 | | 724,052 | |
| 2301 | 60,784 | 14,006 | 2,801 | 43,333 | 7,843 | 1,681 | | 1254,135 | |
| 2333 | 69,106 | 24,390 | 8,672 | 39,759 | 4,878 | 0,542 | | 674,905 | |
| 2375 | 63,721 | 26,977 | 6,512 | 44,737 | 7,442 | 3,721 | | 1361,465 | |
| 2419 | 66,387 | 21,008 | 3,361 | 42,718 | 4,622 | 1,261 | | 723,465 | |
| 2464 | 65,000 | 19,667 | 6,333 | 33,113 | 6,667 | 2,000 | | 1315,681 | |
| 2509 | 76,302 | 13,802 | 2,083 | 36,496 | 0,521 | 0,000 | | 121,464 | |

**Supplementary material legends**

Figure 1: a) Location of the core sampling sites (red stars) on the bathymetric map of the Bay of Brest (source of bathymetric data: https://data.shom.fr). b) Sedimentological descriptions, X-Ray radiographies and photographs of G and KS-02 cores. Map was created using QGIS software version 3.4.5 (https://www.qgis.org).

Figure 2: Age-depth model for the composite “G-KS02” sequence using six 14C-AMS dates for G core (blue code) and eight 14C-AMS dates for KS-02 core (green code) using Bayesian statistics. Vertical lines delimit analysed sections of each core. The age-depth model was made using R 3.5.0 (R Core Team, 2018), the rbacon package^43^.

Figure 3: Detailed pollen diagram as function of depth in the cores G and KS-02 composite sequence. Pollen rates are expressed in percentages compared to the total sum. Only the major taxa are represented (< 2%). The green curve represents the total percentage reached by tree pollen grains (i.e. *Quercus, Alnus, Corylus, Betula, Ulmus, Fagus, Fraxinus, Pinus, Salix*). Anthropogenic pollen indicators used in figure 2 are *Cerealia*-type, *Plantago lanceolata, Rumex, Brassicaceae, Ranunculaceae, Asteroideae and Cichorioideae*. Palynozones (PL for ‘Plougastel peninsula’) were defined based on major changes in pollen percentages of trees, poaceae and *Cerealia*-type. Plalynozone PL-B contains the '1.7–1.4 ka AP event'. ^14^C dates are represented by red stars on the vertical scale.

Table 1: Positions and characteristics of the sediment cores studied.

Table 2: For each 14C date: depth of sampling in the core, dated material, 14C date, error, age calibrated in BP, age calibrated in BC / AD.

Table 3 : Palynological data produced during current study
